# Personalized comprehensive molecular profiling of high risk osteosarcoma: Implications and limitations for precision medicine

**DOI:** 10.18632/oncotarget.5841

**Published:** 2015-10-15

**Authors:** Vivek Subbiah, Michael J. Wagner, Mary F. McGuire, Nawid M. Sarwari, Eswaran Devarajan, Valerae O. Lewis, Shanon Westin, Shumei Kato, Robert E. Brown, Pete Anderson

**Affiliations:** ^1^ Department of Investigational Cancer Therapeutics, Division of Cancer Medicine, The University of Texas MD Anderson Cancer Center, Houston, TX 77030, USA; ^2^ Division of Gynecological Oncology, The University of Texas MD Anderson Cancer Center, Houston, TX 77030, USA; ^3^ Department of Pathology & Laboratory Medicine, The University of Texas-Houston Medical School, Houston, TX 77030, USA; ^4^ Department of Internal Medicine, The University of Texas-Houston Medical School, Houston, TX 77030, USA; ^5^ Department of Orthopedic Oncology, The University of Texas MD Anderson Cancer Center, Houston, TX 77030, USA; ^6^ Department of of Pediatric Hematology/Oncology, Cleveland Clinic Foundation, Cleveland, OH 44195, USA

**Keywords:** personalized medicine, molecular profiling, osteosarcoma, precision medicine, targeted therapy

## Abstract

**Background:**

Despite advances in molecular medicine over recent decades, there has been little advancement in the treatment of osteosarcoma. We performed comprehensive molecular profiling in two cases of metastatic and chemotherapy-refractory osteosarcoma to guide molecularly targeted therapy.

**Patients and Methods:**

Hybridization capture of >300 cancer-related genes plus introns from 28 genes often rearranged or altered in cancer was applied to >50 ng of DNA extracted from tumor samples from two patients with recurrent, metastatic osteosarcoma. The DNA from each sample was sequenced to high, uniform coverage. Immunohistochemical probes and morphoproteomics analysis were performed, in addition to fluorescence *in situ* hybridization. All analyses were performed in CLIA-certified laboratories. Molecularly targeted therapy based on the resulting profiles was offered to the patients. Biomedical analytics were performed using QIAGEN's Ingenuity^®^ Pathway Analysis.

**Results:**

In Patient #1, comprehensive next-generation exome sequencing showed *MET* amplification, *PIK3CA* mutation, *CCNE1* amplification, and *PTPRD* mutation. Immunohistochemistry-based morphoproteomic analysis revealed c-Met expression [(p)-c-Met (Tyr1234/1235)] and activation of mTOR/AKT pathway [IGF-1R (Tyr1165/1166), p-mTOR [Ser2448], p-Akt (Ser473)] and expression of SPARC and COX2. Targeted therapy was administered to match the *P1K3CA*, *c-MET*, and SPARC and COX2 aberrations with sirolimus+ crizotinib and abraxane+ celecoxib. In Patient #2, aberrations included *NF2* loss in exons 2–16, *PDGFRα* amplification, and *TP53* mutation. This patient was enrolled on a clinical trial combining targeted agents temsirolimus, sorafenib and bevacizumab, to match *NF2*, *PDGFRα* and *TP53* aberrations. Both the patients did not benefit from matched therapy.

**Conclusions:**

Relapsed osteosarcoma is characterized by complex signaling and drug resistance pathways. Comprehensive molecular profiling holds great promise for tailoring personalized therapies for cancer. Methods for such profiling are evolving and need to be refined to better assist clinicians in making treatment decisions based on the large amount of data that results from this type of testing. Further research in this area is warranted.

## INTRODUCTION

Osteosarcoma is the most common primary malignant tumor in children, adolescents, and young adults [1]. It comprises a group of mesenchymal bone-forming tumors with multiple histologic subtypes, including osteoblastic, chondroblastic, fibroblastic, and unconventional. The biology of osteosarcoma is complex, and treatment options have remained essentially unchanged for several decades [2]. Since the 1980s, front-line treatment for primary osteosarcoma has consisted of induction chemotherapy followed by surgery for patients with non-metastatic disease. Adjuvant chemotherapy is also given. Options are even more limited for patients with relapsed osteosarcoma. A recent Phase II study of sorafenib and everolimus represents the first positive study for relapsed osteosarcoma in decades, but even this study did not reach statistical significance for the primary outcome [3]. Radium 223 di-chloride, an alpha particle, may be another potential treatment option for patients with osteoblastic metastases [4, 5].

In the era of precision medicine, the molecular profile of a patient's tumor can be determined readily and can be matched to a targeted therapy in real time. This presents an opportunity to systematically analyze an individual patient's tumor and offer them personalized therapy [6]. With few advancements in treatment and the dismal prognosis for patients with relapsed, refractory disease, osteosarcoma patients may benefit from deep molecular, genomic sequencing and comprehensive molecular profiling [2, 6]. As a demonstration of this approach, we performed comprehensive molecular profiling of two patients with metastatic osteosarcoma and matched their profiles to molecularly targeted therapy. Here we report the results of this profiling and discuss the implications and limitations of this approach.

## RESULTS

The patients' clinical histories, molecular profiling results, and targeted therapy are summarized here.

### Patient #1

**Figure 1:**
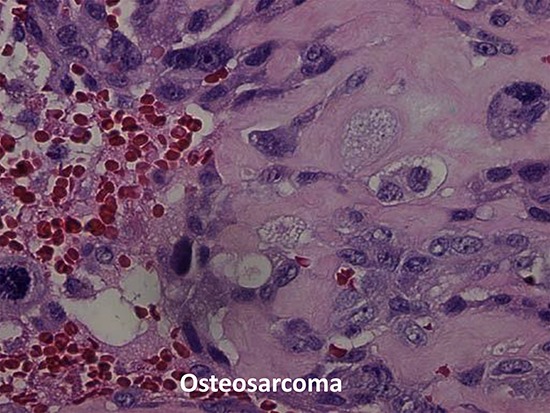
Histopathology figure of osteosarcoma from patient # 1

This 21-year-old male was found to have chrondroblastic osteosarcoma of the right distal femur in 2011. Upon diagnosis, he began treatment with standard first-line chemotherapy comprising methotrexate, doxorubicin, and cisplatin (MAP). He experienced immediate relief from pain with the initial cisplatin and doxorubicin infusions. He then underwent limb salvage surgery, and pathologic review showed his resected tumor to have 80% necrosis. He received postoperative chemotherapy because of his high-risk status and was closely monitored with periodic computed tomographic (CT) scans. Nine months after completion of adjuvant chemotherapy, a routine CT scan showed a 3.5 × 3.5–cm nodule in the left upper lobe of the lung and a 2.0 × 2.5–cm nodule in the right middle lobe, representing distant relapse of the osteosarcoma (Figure 1). Despite treatment with high-dose ifosfamide (1 cycle at 14 g/m^2^), the disease progressed and the patient subsequently developed a pneumothorax. He received another course of cisplatin and doxorubicin, and although the pneumothorax resolved, there was evidence of continued progression of his intrathoracic metastases. He subsequently underwent thoracotomy of the left lung with upper lobe resection and metastectomy. The final pathology report revealed at least 40 foci of tumor in the resected lung tissue. This tumor tissue was sent for comprehensive molecular profiling to identify potential targeted therapy options. The patient was referred to the molecular treatment planning group.

### Patient #1: next-generation exome sequencing

Next-generation sequencing by FoundationOne identified four genomic aberrations: *MET* amplification, *PIK3CA* V344G mutation, *CCNE1* amplification, and a loss of function mutation in *PTPRD* (S1845f*). Our institutional 46-gene panel confirmed the *PIK3CA* V344G mutation. Caris Target One gene profiling revealed positivity for *TLE3* (2+, 80%), androgen receptor (1+, 60%), *TS*, *RRM1*, and *PTEN*. Further testing was negative for *ERCC1*, *MGMT*, *TOPO1*, *ER/PR*, and *SPARC*. FISH analysis revealed no amplification in *Her2/neu* or *TOP2A*. *P1K3CA* and *BRAF* were wild type by sequencing for hotspot alterations. Genetic testing results for Patient #1 are summarized in Table 1 and Table 2.

**Table 1:** Summary of molecular aberrations in genes, receptors, and pathways by various CLIA-certified methods for osteosarcoma Patient #1 and their targeted agents

Detection Method/Therapy	Oncogenic pathway for potential targeted therapy				Chemosensitivity markers	
	*P13K/AKT/mTOR*	*RAS*	*c-MET*	*PTPRD/STAT3*	*SPARC*	*TLE3*
**FoundationOne**	*V334G*		*amplification*	*mutation*		
**Caris Life Sciences**	*WT - V334 G not tested*		*no by FISH*			
**MD Anderson Cancer Center**	*V334G*					
**Morphoproteomics, University of Texas Houston**	*p-mTOR,p-AKT*	*ERK*	*expression*			
**Targeted therapy**	mTOR therapy or P13K	MEK inhibitor	Crizotinib	STAT 3 inhibitor/IGF1R inhibitor	Abraxane	Taxane

**Table 2:** Complete molecular profiling analysis by various CLIA-certified methods for osteosarcoma Patient #1

Marker	Result	Details	Comments
Topoisomerase II alpha	Positive	~70–90% of tumor nuclei (higher percentage of nuclear expression in cellular foci and less in chondroid regions); 26 mitotic figures/10 hpf in cellular regions	Facilitates S to G2 and M phase transitions
Bcl-2	Positive	2+ in cytoplasm of tumor cells	Anti-apoptotic protein
Fatty acid synthase (FASN)	Positive	Highly expressed in cytoplasm (up to 3+)	Tumorigenic protein; correlates with propensity for pulmonary metastasis [62]
P38 mitogen-activated protein kinase (MAPK)	Positive	Expressed to varying degrees in tumor nuclei	Chemoresistance factor; phosphorylated on threonine180/tyrosine182
Excision repair cross complementation group1 (ERCC1)	Positive	Expressed to varying degrees in tumor nuclei	Chemoresistance factor; downstream effector of MAPK
Sirt1 (silent mating type information regulator 2 homolog), NAD+histone deacetylase	Positive	Expressed in 2–3+ in majority of tumor nuclei	Associated with upregulation in response to chemotherapy, mechanism of cancer resistance in human osteosarcoma [63]
Nuclear factor (NF)-kappaB	Positive	0–1/1+, nuclear/cytoplasm	Prosurvival protein, constitutively activated by virtue of expression with nuclear translocation of p-NF-kappaBp65(Ser536)
Signal transducer and activator of transcription (STAT)-3	Positive	0–3+ (majority positive), nuclear	Activated with nuclear expression of p-STAT3(Tyr705) [64, 65]
Cyclooxygenase (COX)-2	Positive	1–3+, cytoplasm	Correlates inversely with survival in patients with osteosarcoma and lung metastasis; highly expressed in cytoplasm of tumor cells, especially more cellular regions [66]
WNT/B-catenin signaling pathway.	Positive	Strong cytoplasmic and nuclear (up to 3+) expression in minor component of tumor cells (cellular foci)	Coincides with nuclear expression of c-Myc protein, whose expression can be upregulated by WNT/B-catenin signaling in ~1/3 of tumor cells [67]; cytoplasmic and/or nuclear staining of Beta-catenin associated in preclinical study and in primary osteosarcomas with lung metastasis [68]
Hypoxia-inducible factor (HIF)-1 alpha	Present	2+ cytoplasmic compartment, without apparent nuclear translocation	Hypoxia-associated protein analyte
HIF-2 alpha, VEGF-A	Present	0–1+ in cytoplasmic compartment	Ischemic-type coagulative necrosis not apparent in the biopsy specimen
Nestin	Present	Strong expression; up to 3+; cytoplasmic aspect	High expression level in osteosarcoma predicted a worse clinical outcome in one study [69]
CD44	Present	Cytoplasmic/plasmalemmal aspect of tumor cells	
Glioma-associated oncogene protein (Gli2)	Present	Variable expression (0–3+) in majority of nuclei	Reflection of either sonic hedgehog pathway signaling or TGF-beta [Smad3] activation of Gli2 [70–72]
Secreted protein acidic and rich in cysteine (SPARC; osteonectin)	Present	Up to 3+ cytoplasmic expression	Predicts poorer relapse and event-free survival rates as well as therapeutic option [73]

### Patient #1: immunohistochemistry based morphoproteomics analysis and therapy

**Figure 2:**
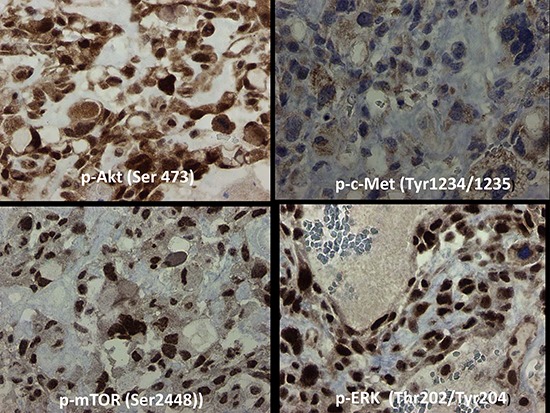
Immunohistochemistry based morphoproteomic studies of osteosarcoma Patient #1 In the tumor cells of Patient #1, c-Met was expressed at 0–1+ (rarely 2+) in the cytoplasm, mTOR at 3+ in nucleus and cytoplasm, ERK 1/2 at 3+ in the nucleus, and Akt at 0–3+ in both nucleus and cytoplasm. Representative sections are shown.

Immunohistochemistry-based morphoproteomic analysis showed constitutive activation of the c-MET pathway in the tumor cells to a mild degree, as evidenced by the expression of phosphorylated (p)-c-Met (Tyr1234/1235) in the cytoplasmic compartment (0 to 1+ with a rare 2+ chromogenic signal on a scale of 0 to 3+) (Figure 2). The insulin-like growth factor (IGF) pathway was expressed with 1+ to 3+ signal in the cytoplasmic compartment of total IGF-1R (Tyr1165/1166). Furthermore, there was moderate expression of PKC-alpha (up to 3+ in both the cytoplasmic and plasmalemmal compartments). The ras/Raf kinase extracellular signal-regulated kinase (ERK) pathway was constitutively activated in the form of phosphorylated p-ERK 1/2 (Thr202/Tyr204). Also present was constitutive activation of the mammalian target of rapamycin C2 (mTORC2) pathway as evidenced by the expression, with nuclear subcellular compartmentalization, of p-mTOR [Ser2448] with concomitant expression of its upstream effector p-Akt (Ser473). The activation of mTORC2 coincides with both downstream signaling from the IGF-1R pathway and strong PKC-alpha expression [7, 8].

**Figure 3:**
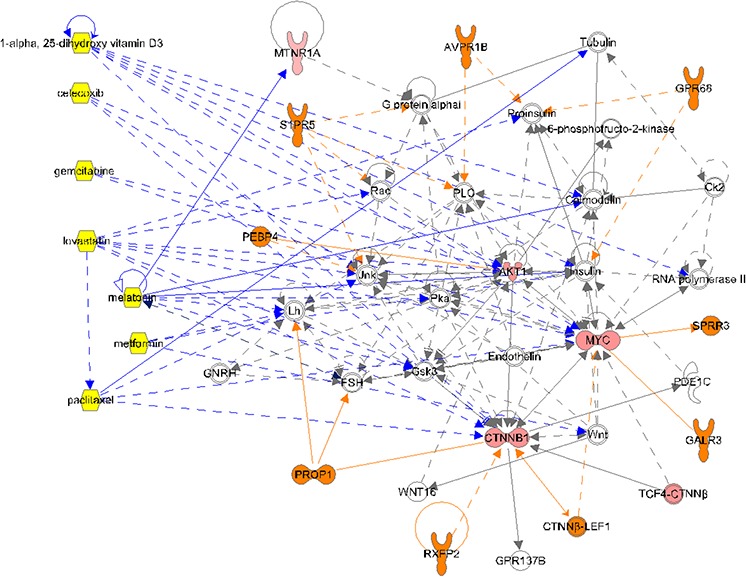
Complex molecular signaling network for osteosarcoma Patient #1 The patient's molecular profiling results were subjected to Ingenuity^®^ Pathway Analysis to identify therapeutic targets. ORANGE: only in disease. PINK: measured analytes (lighter color is lower score). YELLOW: Drugs. DARK BLUE lines: drug interactions.

The biological pathway networks evoked by IPA from the molecular profiles were reviewed for potential targeted therapy options. Of the six biological pathway networks evoked, only one differentiated the patient from the control. In the patient, there were losses of control pathway interactions with insulin and follicle stimulating hormone, and there were gains of interactions with MYC, a key transcription regulator in cancer. Of particular interest was the potentially strong influence of PROP1 on the patient's network, possibly to lower the concentration of beta-catenin (CTNNB1). PROP1 encodes a transcription factor in the pituitary gland. Seven drugs were considered for therapy: 1-alpha25 (OH)2-vitamin D3, celecoxib, gemcitabine, lovastatin, melatonin, metformin, and nab-paclitaxel. As can been seen in (Figure 3), these drugs provided coverage of the patient's pathway network.

Given that the *PIK3CA* V344G mutation was an activating one, Patient #1 was started on metformin and rapamycin therapy, and the response was characterized as stable disease. The *c-MET* amplification seen in the exome sequencing was confirmed by IHC, and thus the patient also received crizotinib, a c-MET/ALK and ROS1 inhibitor. Unfortunately, his disease progressed on the crizotinib, rapamycin, and metformin combination. He was evaluated for another specific c-MET inhibitor study, but that study required a washout period of 4 weeks. Given the aggressive clinical course of his disease, he was started on gemcitabine and nab-paclitaxel therapy on the basis of the SPARC expression identified on morphoproteomic profiling. Celecoxib was added because of the COX2 expression in the IHC specimen. Unfortunately, his disease continued to progress and he eventually died of his disease.

### Patient #2

This 16-year-old male was found to have a tumor in the left proximal tibia with anterior cruciate ligament tear. After biopsy of the tumor, osteoblastic osteosarcoma was diagnosed. The patient received neoadjuvant standard MAP chemotherapy according to the protocol AOST0331 regimen, but he was not officially enrolled in the trial. The patient underwent rotationplasty with surgery that revealed tumor necrosis greater than 95%, with negative margins. He then continued the MAP chemotherapy and developed bilateral hip and groin pain that did not respond to conservative management. An MRI of the left hip suggested a tumor focus, and biopsy showed high-grade metastatic osteosarcoma. The patient began therapy with liposomal doxorubicin and high-dose methotrexate with zoledronic acid. A follow-up positron emission tomographic–CT scan showed progression of disease in the left femur and right hip. He was referred to MD Anderson for treatment on a clinical trial of the alpha emitter radium 223 (http://ClinicalTrials.gov identifier: NCT01833520) [4] and was enrolled on the protocol. After one dose his disease progressed. He then received two cycles of ifosfamide, but his disease continued to progress. The patient underwent left leg amputation due to progressive disease. The patient was presented at treatment planning meeting after he was found to have progression on ifosfamide.

### Patient #2: next-generation sequencing and therapy

Patient #2 had a comprehensive genomic profile that showed loss of *NF2* exons 2–16, *PDGFRA* amplification, C17orf39 amplification, *RB1* loss, and *TP53* loss. The patient's Variant of Unknown Significance report showed the following aberrations: *CUX1* L509V, *GPR124* A1090P, *RAD50* V842A, *FANCD2* amplification, *HGF* R328H, *VHL* amplification, *FGF14* rearrangement, *IL7R* E392K, *FLCN* amplification, and *PCLO* P1027T. The *PDGFR* amplification was confirmed by IHC, showing 90% PDGFRα positive (3+ expression in 90% of tumor cells).

Patient #2 was presented at the molecular treatment planning meeting at MD Anderson. On the basis of his molecular profile, the patient was enrolled in a Phase I clinical trial (http://ClinicalTrials.gov Identifier: NCT01187199) of bevacizumab and temsirolimus in combination with sorafenib for the treatment of advanced cancer [9]. The patient tolerated the therapy reasonably well, with the exception of grade 2 mucositis and grade 1 fatigue. Re-staging scans revealed multiple new bilateral lung opacities with minimal FDG activity, suggesting metastases. There also were new FDG-avid right hilar and left bronchial nodes and a new focus of FDG activity within the left scapula, also suggesting metastasis. Because of progressive disease, he was taken off the clinical trial. The patient elected to receive best supportive care.

## DISCUSSION

Here we report the clinical course, comprehensive molecular profiling, and molecularly matched therapy for two patients with relapsed osteosarcoma. This in-depth analysis of two patients' cases offers insight into the complex biology of high risk osteosarcoma.

In Patient #1, c-*MET* amplification was shown by exome sequencing, and there was evidence that downstream activation of related pathways contributed to the growth of this patient's tumor. *MET* overexpression has been demonstrated to convert primary human osteoblasts into osteosarcoma [10]. The Met/HGF receptor has been shown to be overexpressed in human osteosarcomas and is activated by either a paracrine or an autocrine circuit, and therefore might play a role in aggressive osteosarcomas [11]. HGF activates both the mitogen and “motogen” machinery in human osteosarcoma cell lines, and this is related to activation of the ERK and Akt pathways [12]. P-ERK 1/2 (Thr202/Tyr204) and p-Akt (Ser473) were expressed in this patient's tumor (Figure 2). A small-molecule Met inhibitor, PF2362376, inhibited phosphorylation of Met, Erk, and Akt while inhibiting the proliferation of canine osteosarcoma cell lines and induced cell death at biologically achievable concentrations, and further was found to block activities associated with Met signaling, including migration, invasion, branching morphogenesis, and colony formation in soft agar [13].

In the morphoproteomic laboratory, the phospho-specific probe for tyrosine 1234/1235 was used to assess the state of phosphorylative activation of c-Met in Patient #1′s tumor cells; it detected 0 to 1+ (rare 2+) staining intensity on a scale of 0 to 3+. This is a relatively low level of expression of the constitutively activated c-Met pathway. The Caris profile, furthermore, did not show c-*MET* amplification by FISH. One of the reasons could be tumor heterogeneity.

The *PIK3CA* missense mutation V344G was detected in Patient #1′s tumor by exome sequencing by two different laboratories. This may represent an activating mutation and thus contribute to PI3K/Akt/mTOR pathway signaling. This was further reflected in the constitutive activation of the mTOR signal transduction pathway in the tumor. The PI3K/Akt/mTOR pathway has been reported to contribute to osteosarcoma progression [14]. The confirmed presence of *MET* amplification and p-ERK and p-AKT by IHC prompted the patient's treatment with crizotinib and rapamycin, but unfortunately he did not have a significant clinical response to this combination.

The *CCNE1* amplification identified in Patient #1′s tumor by FoundationOne next-generation sequencing is important in that it encodes the cyclin E1 protein (which regulates G1 to S phase transition) via binding to and activating cyclin-dependent protein kinase 2 (cdk2). This protein complex has a direct role in initiation of replication and overall maintenance of genomic instability [15]. This particular amplification was reported in 27% of osteosarcomas (6/22) in one study, and was directly correlated with increased cyclin E1 expression, but it does not correspond with chemotherapy response rates [16]. There are no approved therapies that target the *CCNE1* amplification, but clinical trials of cdk2 protein inhibitors are underway.

The *PTPRD* S1845fs*2 mutation detected in Patient #1′s tumor is an altered tumor suppressor gene that dephosphorylates the oncoprotein Stat3 [17], thus inactivating it. There are no approved treatments or clinical trials focused on *PTPRD*. However, the increased Stat3 activity induced by this mutation is a potential target, as clinical trials of inhibitors of Stat3 are underway. Interestingly, somatic and germline *PTPRD* mutations have been noted in Ewing sarcoma, another bone sarcoma that occurs in children and young adults. In a small study that sequenced the tumors of a few patients, it was intriguing that *PTPRD* germline aberrations conferred clinical benefit to patients with Ewing sarcoma who received therapy directed at the IGF1R and mTOR pathway [18].

After failure of crizotinib and rapamycin in Patient #1, additional morphoproteomic analysis was done that showed SPARC and COX2 expression. The patient then was started on a combination of gemcitabine, nab-paclitaxel, and celecoxib on the basis of these findings. Nab-paclitaxel binds to tubulin and stabilizes it, leading to mitotic catastrophe and apoptosis [19, 20]. By stabilizing beta-tubulin, the taxanes sequester Smad3 and reduce TGF-beta signaling [20]. Nab-paclitaxel's uptake is facilitated by SPARC [21–23], which was highly expressed in the patient's tumor, suggesting that the tumor may have been sensitive to this cytotoxic agent. In a xenograft model of human osteosarcoma with high SPARC expression, nab-paclitaxel showed a tumor inhibition rate of 98.8%, which was greater than those of doxorubicin (46.1%) or paclitaxel (40.8%) [24].

The sensitivity of tumor cells to taxanes can be enhanced by metformin via downregulation of p38MAPK-dependent ERCC1. Metformin inhibits both the mTORC1 pathway and components of the mTORC2 pathway (expressed in this patient's tumor) by activating AMPK [25]. It also inhibits the tumorigenic FASN pathway [25, 26], which was expressed in Patient #1′s tumor and has previously been associated with aggressive lung metastasis in osteosarcoma [27]. *In vitro*, metformin inhibits the mevalonate pathway and leads to increased cytotoxicity of anticancer drugs in an additive manner against human osteosarcoma cells [28]. It decreases the capacity of advanced glycation end products to upregulate the expression of RAGE in osteoblastic cells [29] and thereby could contribute to downregulation of the tumorigenic ERK pathway [30]. This is relevant to the observation that S100A7 promotes the migration and invasion of osteosarcoma cells via the receptor for advanced glycation end products [31].

In spite of the interrelated targetable pathways in Patient #1′s tumor, his cancer continued to progress. The lack of clinical benefit of the targeted therapy combinations may be attributable to the identified aberrations not being the driver aberrations or to the drugs chosen off-label not being the best ones to target the aberrations. Perhaps a specific P13K inhibitor or a more potent c-MET inhibitor could have yielded a more favorable response. These suggestions are speculative, but they reflect the complex biology of relapsed osteosarcoma biology, which may entail activation of multiple drug resistance pathways.

In Patient #2, the potential actionable aberrations included *NF2* loss and *PDGFRA* aberration. Merlin, encoded by *NF2*, functions by coordinating signaling of receptor tyrosine kinases, such as the epidermal growth factor receptor (EGFR), with cell contact; the inactivation of Merlin in cancer disrupts this mechanism and leads to unrestrained receptor tyrosine kinase signaling despite cell contact [32]. Any alteration that results in the partial or complete loss of the FERM domain (amino acids 22–311) or the NF2 *C*-terminal moesin domain (aa 532–595) is predicted to cause a loss of function [33, 34]. Therefore, the alteration observed in this tumor is expected to cause a loss of function. The *NF2* mutation was not reported in 14 osteosarcomas analyzed (COSMIC, Aug 2014). Mice heterozygous for *NF2* develop a variety of cancers, including osteosarcomas [35], although neurofibromatosis 2 patients do not normally develop osteosarcomas, nor are mutations in NF2 frequently found in human osteosarcoma samples [36]. At present, there are no approved therapies that directly target *NF2* loss. However, preclinical studies in models of *NF2* loss have suggested that the mTOR inhibitors may be a relevant approach [37]. The mTOR inhibitors everolimus and temsirolimus have been approved by the U.S. Food and Drug Administration (FDA) for other cancer types and are under clinical investigation in solid tumors [38].

*PDGFRA* encodes platelet-derived growth factor receptor alpha (PDGFR-alpha), a tyrosine kinase receptor that, upon binding of cognate ligands (PDGFA or PDGFB), activates several signaling pathways, including PI3K and MAPK11. *PDGFR* aberrations, including point mutations, translocations, amplification, and/or overexpression, have been associated with various malignancies, leading to consideration of their protein products as oncoproteins [39]. *PDGFRA* alterations have not been reported in any of the 54 osteosarcoma cases analyzed (COSMIC, Nov 2014). PDGFR-alpha expression was reported in 4–90% of osteosarcomas, but its expression was not correlated with overall or disease-free survival [40, 41]. Several tyrosine kinase inhibitors that target the PDGFR-alpha and Beta proteins, as well as other kinases, are FDA-approved for other indications. These agents, including imatinib, sunitinib, sorafenib, dasatinib, nilotinib, regorafenib, ponatinib, and pazopanib, are currently in clinical trials for patients with various solid tumor types.

*PDGFRA* activation leads to activation of the PI3K/Akt and mTOR pathways [42]. Therefore, PI3K and mTOR pathway inhibitors may be relevant in a tumor with PDGFRA amplification. The mTOR inhibitors everolimus and temsirolimus are FDA-approved for other indications and remain under investigation in multiple solid tumor types.

Functional loss of the tumor suppressor p53, which is encoded by the *TP53* gene, is common in aggressive advanced cancers [43]. Germline mutations in *TP53* are associated with the very rare Li-Fraumeni syndrome and the early onset of many cancers, including osteosarcoma [44]. *TP53* mutations have been reported in 27% of osteosarcomas (COSMIC, October 2014). Interestingly, it has been shown that, among patients with advanced cancer, those whose tumor had a *TP53* mutation had a longer progression-free survival interval on a bevacizumab-containing regimen than those whose tumor had a wild-type *TP53* [45].

These aberrations directed us to choose for Patient #2 a clinical trial of the combination of sorafenib, bevacizumab, and temsirolimus. Even this targeting of multiple pathways with a cocktail of drugs, however, was not able to overcome the complex biology of osteosarcoma. mTOR inhibitors were used in combination with other targeted therapies. It has been shown that mTOR inhibitors can overcome primary and/or acquired resistance to various tyrosine kinase inhibitors and monoclonal antibodies in various malignancies [9, 18, 46, 47].

Cancers like melanoma have frequent aberrations of the *BRAF* gene in more than 50% of patients and have three US FDA approved drugs targeting the *BRAF V600 E* aberration. Non-small cell Lung cancer is now a heterogenous disease with *EGFR*, *BRAF*, *Her2/Neu* aberrations or *ALK, ROS1, RET* or *FGFR* fusions [48–52]. These cancers are examples of recurrent driver aberrations with tyrosine kinase inhibitors in the clinic that are useful to act upon these genes. Even in these cancers although these genes are considered dominant drivers and frequently have exceptional responses to kinase inhibitors, resistance eventually develops. In fact, resistance mutations have been described with every kinase inhibitor [53]. Resistance mechanisms are complex and can be innate and / or acquired in the same kinase domain or through another mechanism upstream or downstream [18, 54]. Relapsed osteosarcoma is a highly aggressive disease and has an extremely complex biology. The clinical next generation sequencing and the IHC based profiling have their own limitations in terms of the genes or proteins tested. Firstly osteosarcoma is a disease that bears the stamp of chromothripsis arising from a single cellular catastrophic event than accumulation of aberrations over time. The aberrations that arise from this crisis are thus unpredictable other than *TP53* which is till date one of the undruggable genes [55]. Secondly, the patients were given targeted therapy after chemotherapy refractory disease when the biology of the tumor and the drug resistance mechanisms are complex. Thirdly, we did not have active drugs in the clinic against several of the proteins tested eg: WNT pathway inhibitor or Glioma-associated oncogene protein (Gli2) pathway inhibitor. Fourthly, there is intra-tumoral and inter-tumoral heterogeneity. The metastatic sites of the disease would probably have a different biology when compared to the primary and secondary sites which was profiled. Lastly, there was a recent study published that showed the lack of efficacy of molecularly targeted agents outside of their approved indications did not improve progression-free survival when compared to physicians choice in advanced cancer patients [56]. So it may be that it may have not been the right drug to the right target at the right time and single kinase inhibitors may work only in rare cancer subsets. Moreover, most of the hallmarks of cancer are controlled by signaling networks at the cellular level that need a strategy combing kinomics, proteomics, drug screening with novel approaches using a comprehensive translational approach in appropriate models including patient derived xenograft models, genetically engineered mouse models and co-culture systems, more than the clinical next generation sequencing and IHC based approaches we have used.

One of the limitations of this study is that the patients were not tested for programmed cell death ligand 1 (PDL1) expression. PDL1 was recently shown to correlate with tumor-infiltrating lymphocytes in a subset of osteosarcoma patients [57, 58]. Given the increasing portfolio of agents targeting PD-1/PDL1, targeting this immune checkpoint may be an option for osteosarcoma.

## MATERIALS AND METHODS

We identified two patients with relapsed, metastatic osteosarcoma who presented for targeted therapy options to the Clinical center for targeted therapy at The University of Texas MD Anderson Cancer Center. Their clinical information was obtained through review of their medical records. Treatment, consent and patient treatment on targeted therapy or on the investigational trial and data collection were performed in accordance with the guidelines of The University of Texas MD Anderson Cancer Center Institutional Review Board (IRB).

### Molecular profiling of tumor samples

The patients' tumor samples were subjected to molecular profiling by next-generation exome sequencing of 186 cancer genes by the Illumina platform (FoundationOne, Boston, MA); by “Caris Target Now”–based molecular profiling (Caris Life Sciences, Irving, TX) that included immunohistochemistry (IHC), fluorescence *in situ* hybridization (FISH), and sequencing; by a Clinical Laboratory Improvement Amendments (CLIA)–certified MD Anderson Cancer Center next-generation sequencing 46-gene panel. In addition immunohistochemistry and morphoproteomics analyses were performed. These methods have been previously published [18, 59–61].

### Biomedical analytics

The case scales from the morphoproteomic analysis were weighted and normalized to a set of profile scores according to a biomedical analytics algorithm customized for the pathologist. The scores, along with the Uniprot IDs of the measured analytes, were analyzed through the use of QIAGEN's Ingenuity^®^ Pathway Analysis (IPA^®^, QIAGEN Redwood City, http://www.qiagen.com/ingenuity) to evoke the most likely biological pathways. A “Control” profile, consisting of the same Uniprot IDs without scores, was also created to minimize search bias in the IPA data mining. The patient's evoked pathway networks were then compared with those of the control, and evoked molecules unique to the tumor were identified. The interactions of proposed drugs with the identified disease-related biological pathway network(s) were then highlighted.

## CONCLUSION

We report a comprehensive genomic profile and proteomic analysis of two patients with recurrent, metastatic osteosarcoma. Although potential targetable genomic alterations were identified in both tumors, these patients did not derive clinical benefit from the treatments chosen to target their cancer. Osteosarcoma is a heterogenous disease with a high degree of intratumoral variability. Although there is great promise in personalized medicine, these cases highlight the need for alternative, more effective approaches to guiding treatment decisions in this disease given the large volume of data generated by molecular profiling. Future measures exploring the unique biology and molecular pathogenesis of rare tumors may pave the way for the development of more specific and effective targeted therapies.
